# Comparative experimental infection of *Listeria monocytogenes* and *Listeria ivanovii* in bovine trophoblasts

**DOI:** 10.1371/journal.pone.0176911

**Published:** 2017-05-03

**Authors:** Cláudia E. Rocha, Juliana P. S. Mol, Luize N. N. Garcia, Luciana F. Costa, Renato L. Santos, Tatiane A. Paixão

**Affiliations:** 1 Departamento de Patologia Geral, Instituto de Ciências Biológicas da Universidade Federal de Minas Gerais, Belo Horizonte, Brazil; 2 Departamento de Clínica e Cirurgia Veterinárias, Escola de Veterinária da Universidade Federal de Minas Gerais, Belo Horizonte, Brazil; University of Colorado Denver School of Medicine, UNITED STATES

## Abstract

*Listeria monocytogenes* is a Gram-positive, facultative intracellular and invasive bacterium that has tropism to the placenta, and causes fetal morbidity and mortality in several mammalian species. While infection with *L*. *monocytogenes* and *L*. *ivanovii* are known as important causes of abortion and reproductive failure in cattle, the pathogenesis of maternal-fetal listeriosis in this species is poorly known. This study used the bovine chorioallantoic membrane explant model to investigate the kinetics of *L*. *monocytogene*s, *L*. *ivanovii*, and *L*. *innocua* infections in bovine trophoblastic cells for up to 8 h post infection. *L*. *monocytogenes* and *L*. *ivanovii* were able to invade and multiply in trophoblastic cells without causing cell death or inducing expression of pro-inflammatory genes. Although *L*. *innocua* was unable to multiply in bovine trophoblastic cells, it induced transcription of the pro-inflammatory mediator CXCL6. This study demonstrated for the first time the susceptibility of bovine trophoblastic cells to *L*. *monocytogenes* and *L*. *ivanovii* infection.

## Introduction

*Listeria monocytogenes* is a facultative intracellular Gram-positive bacterium that causes an important zoonotic disease often associated with foodborne epidemics [1]. There are seventeen recognized *Listeria* species, but only *L*. *monocytogenes* and *L*. *ivanovii* have been reported as important causative agents of the disease [2, 3]. *L*. *monocytogenes* is considered the most pathogenic species, and it is more often associated with disease in ruminants and humans [1, 4]. In humans, listeriosis has various clinical manifestations, predominantly in immunocompromised patients. Human *L*. *monocytogenes* infections may be associated with sepsis, encephalitis, organ-restricted infections such as endocarditis and hepatitis, maternal-fetal infection, and self-limiting gastroenteritis [5].

*L*. *monocytogenes* infection in cattle is mainly associated with meningoencephalitis and abortion, but it may also result in neonatal septicemia, mastitis, and keratitis [2]. *L*. *ivanovii* infection is associated with fetal death, stillbirths and premature births in ruminants, although it is less common than *L*. *monocytogenes* [2]. *L*. *ivanovii* causes bovine abortion, but has not been associated with meningoencephalitis in ruminants, and it rarely infects humans causing bacteremia, fetal loss, or gastroenteritis [6]. Although sporadic, bovine listeriosis can cause significant economic losses due to reproductive failure and mortality. In experimental infections, ovine and bovine pregnant uteruses are considered highly susceptible to *Listeria* sp., which is associated with abortion and fetal infection [2]. *Listeria* spp. infection in pregnant ruminants causes necrotic placentitis and abortion, which occurs predominantly during the last third of gestation, and infected fetuses usually develop autolysis, bronchopneumonia, hepatitis, and necrotizing splenitis [2]. *Listeria* spp. can be isolated from both the placenta and fetus [2], and it may also be cultured from milk samples and the mammary gland of lactating cows [7].

Both *L*. *monocytogenes* and *L*. *ivanovii* can multiply in phagocytic and non-phagocytic cells *in vitro* [6, 8, 9, 10, 11, 12, 13, 14, 15]. Several virulence factors that play roles in invasion, proliferation, and cell to cell dissemination have been identified [16]. Transcriptional activator PrfA regulates expression of four major of virulence factors involved in host infection, namely InlA, InlB, LLO, and ActA. Proteins InlA and InlB that mediate host cell entry, LLO that mediate phagosome escape, and ActA that mediate actin based movement and cell-to-cell spread [16]. Previous studies have demonstrated the importance of these virulence factors, in the establishment of intrauterine and fetal infection by epidemiological evidence in human patients [17] or by using laboratory animal models [16, 18, 19].

While listeriosi*s* is known as an important cause of abortion and reproductive failure in cattle, its maternal-fetal pathogenesis is poorly known. Previous studies addressing the pathogenesis of *Listeria* spp. have used human placental explants or trophoblastic cell lines [11, 16, 20, 21] or laboratory animals [19, 22, 23, 24, 25]. Importantly, human and bovine placental tissues have marked morphologic and physiologic differences, which prevent a direct extrapolation of data generated with human placental tissues to the pathogenesis in pregnant cows. Similarly, the mouse model is a suitable experimental infection model, but it is equally inadequate for modeling *Listeria*-induced bovine placentitis. Interaction of bovine trophoblast with intracellular pathogen was studied [26]. Therefore, experimental infections of bovine placental explants with *Listeria* spp. *in vitro* may greatly contribute to a better understanding of the pathogenesis of listeriosis in cattle. Thus, this study aimed to evaluate the interaction of *Listeria* spp. with bovine trophoblastic cells determining kinetics of infection and cellular responses in a bovine chorioallantoic membrane (CAM) explant model.

## Materials and methods

### Bovine CAM explants

Bovine pregnant uteruses at the middle and final thirds of gestation were obtained at a local slaughterhouse (Distribuidora de Carnes Sabara Eireli, Sabará, Minas Gerais, Brazil) for preparation of CAM explants. Gestational ages were estimated by measuring fetal crown-rump length as previously described [27]. Experimental procedures were approved by the Ethics Committee in Animal Experimentation of Universidade Federal de Minas Gerais (CEUA/UFMG, protocol 76/2012) CAM explants were prepared as previously described [26] with modifications. The pregnant uterus was opened under aseptic conditions and macroscopic evaluation of the placenta and fetus was performed. Edema, exudate, hemorrhage, and adventitial placentation were evaluated. Placentas without gross changes were incised to remove the CAM, which was immediately washed in a MEM medium (Life Technologies, USA) with 50 U/mL of penicillin and 50 μg/mL of streptomycin (Pen Strep, Gibco, USA) for 20 min. CAM were washed two times with MEM without antibiotics, mounted onto a support, and placed into six-well cell culture plates with supplemented medium (DMEM F12 with 10% fetal bovine serum, 1% pyruvate and 1% essential amino acids) in contact with the trophoblastic surface. The support used in the preparation of the explants were cleaned and sterilized in 3% hypochlorite for 24 h, followed by washing in E-toxaclean (Sigma-Aldrich, USA) for 24 h, followed by absolute alcohol, and distilled water rinses, and finally autoclaved. Fragments of placenta were collected for DNA detection of *Listeria* spp. by multiplex PCR [28]. Only placentas that were negative by PCR were used in experiments.

### Bacterial strain and growth conditions

*L*. *monocytogenes* (ATCC 7644), *L*. *ivanovii* (ATCC 19119), and *L*. *innocua* (ATCC 33090) were used in this study. They were grown in 20 mL of BHI broth (brain heart infusion, Kasvi, USA), at 37°C for 15 h under agitation (200 rpm). Bacterial culture was centrifuged at 3000 g for 10 min at room temperature, and ressuspended in phosphate buffered saline (PBS). Optical density OD600 of bacterial suspensions was measured by spectrophotometry. The inoculum was prepared in supplemented DMEM F12 medium.

### CAM explants infection

A volume of 200 μL of supplemented DMEM F12 medium containing 1 x 10^6^ bacteria/mL was added to the trophoblastic surface of the CAM in each well, corresponding to a multiplicity of infection of 10 bacteria per cell (MOI 1:10), considering the average of 20,000 trophoblasts by explant, as previously described [26]. Non-infected explants were used as controls. Soon after inoculation, plates were centrifuged at 400 g for 10 min at 22°C, and maintained at 37°C with 5% CO_2_ for 30 min to allow for bacterial internalization. The supernatant was then removed from each well, and replaced with 200 μL of sterile supplemented DMEM/F12 containing gentamycin 50 μg/mL (Gibco, USA). The plates were incubated at 37°C for 1 h to inactivate extracellular bacteria. Then, the wells corresponding to later time points (i.e. 4 and 8 h post infection—hpi) had the media replaced by fresh media containing 25 μg/mL gentamycin. To determinate infection kinetic of *Listeria* spp., the supernatant was removed at 1, 4, and 8 hpi, and CAM explants were washed once with PBS, followed by lyses of trophoblastic cells with 200 μL 0.1%Triton X-100 (Roche, Germany) for 10 min. The recovered in 200 μL of sterile PBS and diluted in 1 mL. The serial dilutions were plated on BHI agar (Kasvi, USA) and maintained at 37°C for 24 h for CFU (colony forming units) counting. Bacterial intracellular invasion was showed at 1 hpi and intracellular survival was showed at 4 and 8 hpi. Eight hours have been enough to study kinetic of *Listeria* sp. infection in culture of epithelial cells [9, 29]. Preliminary experiments were performed comparing the invasion and intracellular survival of different multiplicity of infection of *L*. *monocytogenes* per cell (MOI 1, 10 and 100) at 1, 4 and 8 hpi as detailed above using 3 placentas per MOI.

### Histology and immunohistochemistry

CAM explants (uninfected or infected with *L*. *monocytogenes*) were collected at 1, 4, and 8 hpi, and fixed in 10% buffered formalin for 24 h, followed by paraffin embedding. Three μm sections were stained with hematoxylin and eosin (HE). Immunohistochemistry was performed to confirm intracellular localization of *L*. *monocytogenes i*n trophoblasts. CAM sections were deparaffinized, hydrated, and incubated twice with 4% hydrogen peroxide in PBS for 45 min, followed by incubation with skim milk (1:10 dilution) as a blocking solution for 60 min, and then incubated with a polyclonal anti-*L*. *monocytogenes* primary antibody (Listeria O Poly Antiserum serotypes 1,4, Difco, USA) at 1: 100 dilution with 1% bovine serum albumin for 45 min, in a humidified chamber at room temperature. Slides were washed in PBS and incubated with biotinylated secondary antibody for 30 min at room temperature, washed in PBS again, and then incubated with streptavidin-peroxidase complex (LSAB kit; DAKO Corporation, USA) for 30 min at room temperature. The reaction was revealed with AEC solution (Dako) for 10 min, and sections were counterstained with Mayer’s hematoxylin.

### Cell mortality analysis

Cell mortality rate was analyzed by measuring lactate dehydrogenase (LDH) release in the supernatant from uninfected or infected CAM explants. Supernatants were collected at 1 and 8 hpi and stored at -80°C for LDH dosage using the CytoTox 96 Non-Radioactive Cytotoxicity Assay kit (Promega, USA). Briefly, 50 μL of each sample (1:10 dilution) were placed in a 96 well plate, 50 μL of the mix substrate reagent as added and incubated for 15 min, followed by addition of 50 μL of stop solution. After 60 min, the quantification was performed at 490 nm using an ELISA reader (Epoch Bioteck, USA). The overall mortality of each CAM explant was obtained by lysis of trophoblasts with substrate mix (CytoTox 96) according to the manufacturer’s protocol. The optical density obtained with this treatment was considered 100% of cell mortality.

### Real time quantitative PCR analysis

CAM explants of the third trimester of pregnancy infected with *Listeria* spp. were used for RNA extraction and assessment of pro-inflammatory and immunomodulatory gene transcription, namely: CXCL6, CCL2, interleukins IL-8, IL-6, tumor necrosis factor alfa (TNFα), and interferon tau (IFNτ). GAPDH and 18S were used normalizing genes. Customized primers and probes FAM-MGB were commercially available (TaqMan Arrays Fast 96 well Plates Applied Biosystems, USA), and used for TaqMan (Applied Biosystems, USA) assessment of transcription of CXCL6 (Bt03259300_m1), CCL2 (Bt03212321_m1), IL-8 (Bt03211906_m1), IL-6 (Bt03211905_m1), TNFα (Bt03259154_m1), IFNτ (Bt03210579_g1 GAPDH (Bt03210913_g1), and 18S (Hs99999901_s1). The primers and probes were customized for the Taqman gene expression system (Applied Biosystems, USA). Total RNA from bovine CAM explants was extracted at 1 and 4 hpi, time points with similar intracellular CFU for all *Listeria* species studied. RNA extraction was made with Trizol Plus (Invitrogen, USA), and stored at -80°C. One μg of total RNA was treated with DNase (Invitrogen, USA) according to the manufacturer's instruction. The quantity and quality of the total RNA was assessed by spectrometry 260/280 ration and agarose gel electrophoresis. Reverse transcription was performed using 200 ng of total RNA and Taqman reverse transcription kit (Applied Biosystems, USA) according to the manufacturer’s instructions. Quantitative PCR was performed for each sample in duplicate using 2 μL of cDNA in 20 μL reaction of II TaqMan Universal Master Mix (Applied Biosystems, USA) in the thermal cycler Step One plus (APPLIED BIOSYSTEMS, USA) according to the manufacturer's instructions. Data were analyzed using the comparative threshold cycle (CT) method [30] (S1 Fig). The CT values were normalized based on the expression of GAPDH.

### Statistical analysis

After logarithmic transformation, CFU and gene transcription data were subjected to ANOVA and compared between groups by the Student–Newman–Keuls test. Cell mortality rate, calculated as a percentage, was analyzed by non-parametric tests Mann Whitney or Kruskal-Wallis test. Statistical analyses were performed using GraphPad Prism 5 (Graphpad Software, USA). The results were considered significant when p<0.05.

## Results

### Infection of bovine trophoblastic cells with *L*. *monocytogenes*

**Fig 1:**
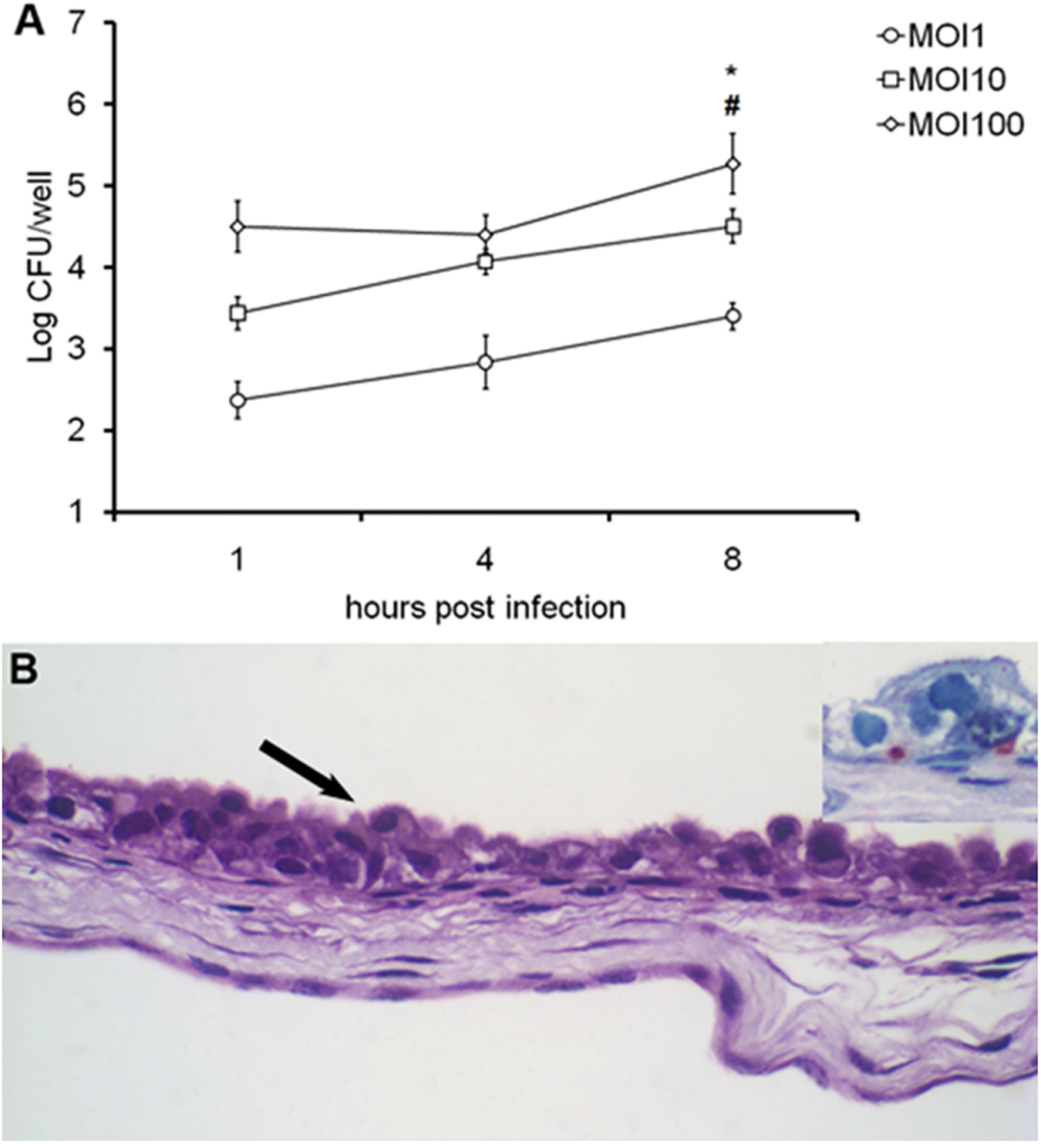
*Listeria monocytogenes* infection of bovine chorioallantoic membrane (CAM) explants. (A) CAM explants inoculated with variable MOI (1, 10, or 100) of *L*. *monocytogenes*. CFU numbers of intracellular bacteria were measured at 1, 4 and 8 hpi. Data points represent means and standard errors of three independent experiments performed in triplicate (One-way ANOVA, * p <0.05, significant difference between 1 and 8 hpi in the same MOI; and ^#^ p <0.05, significant difference between different MOI in time 8 hpi). (B) Infected CAM, and immunostained *L*. *monocytogenes* in red (in detail) in infected trophoblastic cells at 4 hpi. Trophoblastic cells are indicated by arrow. HE.60X.

Considering that there are no previous reports of *in vitro* infection of bovine trophoblastic cells with *L*. *monocytogenes*, we initially evaluated the kinetics of infection of CAM explants obtained at the last trimester of pregnancy with variable MOI (1, 10 and 100). Nine placentas third trimester (three per MOI) were used in this experiment. *L*. *monocytogenes* invasion of trophoblastic cells was proportional to the MOI. Significant differences in CFU numbers were observed with a MOI of 10 when comparing 1 to 8 hpi (Fig 1A). Based on these results, all additional experiments were performed with MOI of 10. Importantly, the model was further characterized by morphological analysis and immunostaining for assessment of intracellular localization of *L*. *monocytogenes* in trophoblasts of CAM explants (Fig 1B) confirming the viability of this model to study the interaction of *Listeria* spp. with bovine trophoblasts *in vitro*.

**Fig 2:**
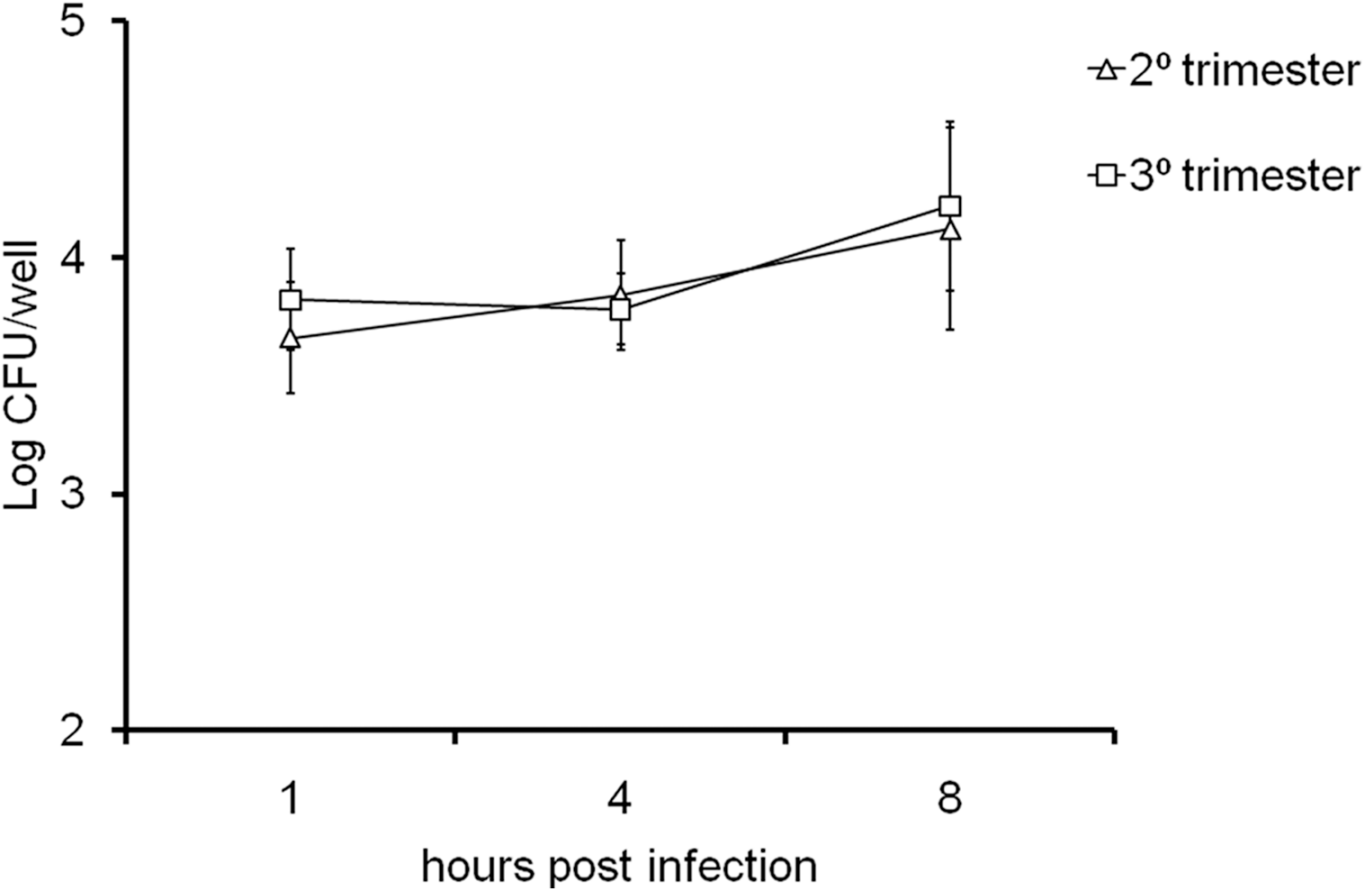
Kinetics of *L*. *monocytogenes* infection in CAM explants derived from bovine placentas at the second and third trimesters of pregnancy. CAM explants were inoculated with 1x 10^6^ CFU/mL of *L*. *monocytogenes*, and intracellular bacteria were measured at 1, 4, and 8 hpi. Data points represent means and standard errors of five independent experiments per group performed in triplicate. There was no statistically significant difference.

*Listeria*-induced abortion in cattle is commonly observed during the third trimester of pregnancy [2]. Therefore, we compared the kinetics of *L*. *monocytogenes* infection in CAM explants obtained at the second or third trimester of pregnancy. Five placentas of cows in the second (3.4 to 4.8 months) and five in the third trimester (7.1 to 8.8 months) were used in this experiment. The number of *L*. *monocytogenes* CFU recovered from bovine CAM explants at 1, 4, and 8 hpi were similar when comparing the second and the third trimesters of gestation (Fig 2). These results demonstrate that *L*. *monocytogenes* invades and multiplies in the trophoblastic cells regardless of the gestational stage.

### Infection of bovine trophoblasts with different species of *Listeria* spp.

**Fig 3:**
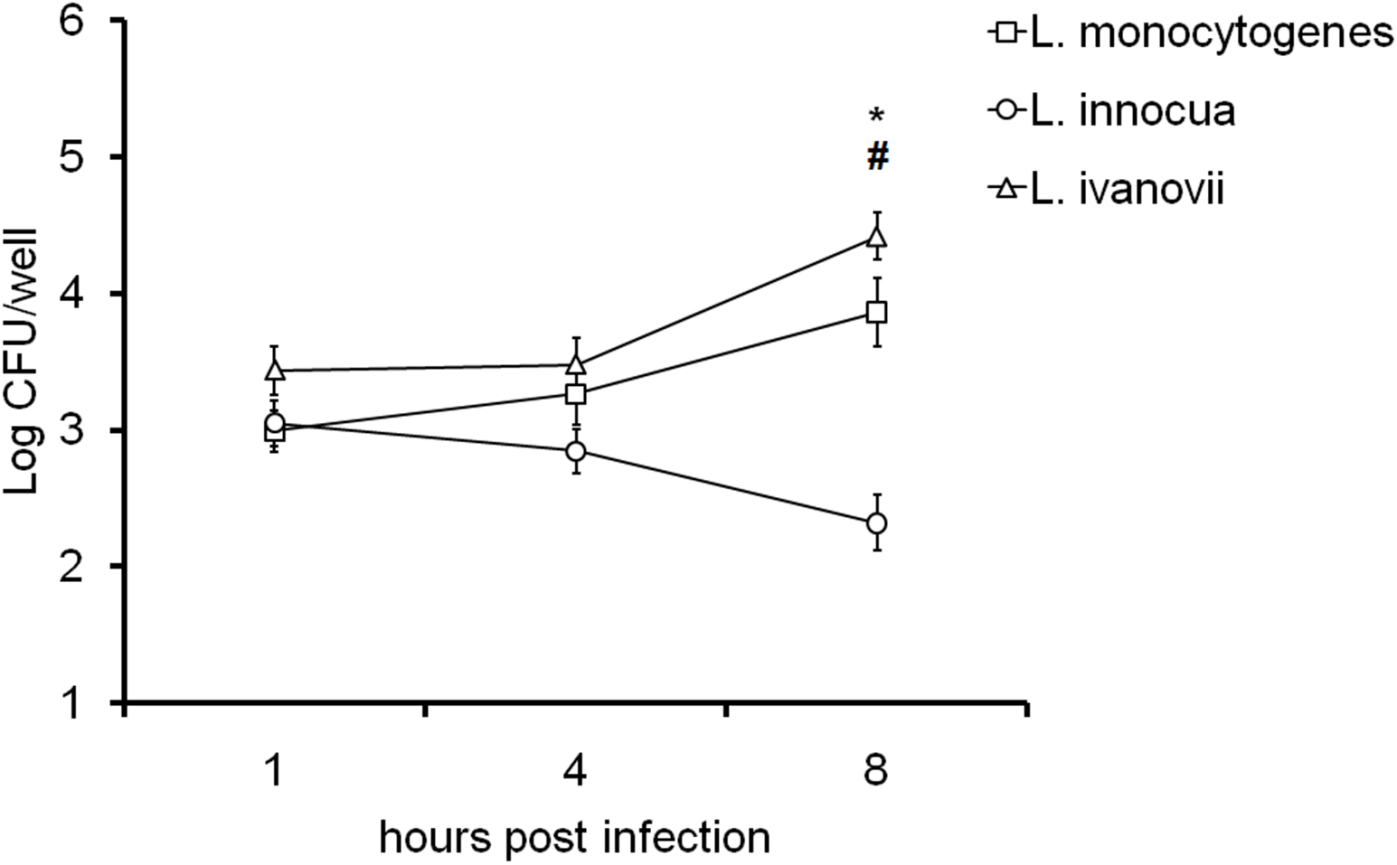
Bovine CAM explant infections with different *Listeria* spp. CAM explants were inoculated with 1x 10^6^ CFU/mL of *L*. *monocytogenes*, *L*. *innocua*, or *L*. *ivanovii*, and intracellular bacteria were measured at 1, 4, and 8 hpi. Data points represent means and standard errors of five independent experiments performed in triplicate (One-way ANOVA; * P <0.05, significant difference between the same species of *Listeria* sp. at 1 h and 8 hpi; and ^#^ P <0, 05; significant difference between *L*. *innocua* and other species at 8 hpi).

Similar to *L*. *monocytogenes*, *L*. *ivanovii* is known to cause abortion in ruminants [2]. Conversely, *L*. *innocua* is considered a non-pathogenic species [18]. Thus, invasion and multiplication of different species of *Listeria* were evaluated in CAM explants infected with *L*. *monocytogenes*, *L*. *innocua*, or *L*. *ivanovii*. There was no difference in CFU numbers recovered of different *Listeria* spp. at 1 hpi, indicating that there was no difference in the invasion of bovine trophoblast cells by these three species (Fig 3), even when CFU data at 1 hpi were normalized according to the CFU numbers in the inocula from each *Listeria* species (data not shown). However, a significant increase in CFU numbers of *L*. *monocytogenes* and *L*. *ivanovii* recovered from CAM explants was observed at 8 hpi, whereas there was a reduction in CFU numbers of *L*. *innocua* under the same conditions and at the same time point. CFU numbers of *L*. *monocytogenes* and *L*. *ivanovii* were approximately 1 log higher than those of *L*. *innocua* at 8 hpi, indicating that *L*. *innocua* was unable to multiply in bovine trophoblastic cells.

**Fig 4:**
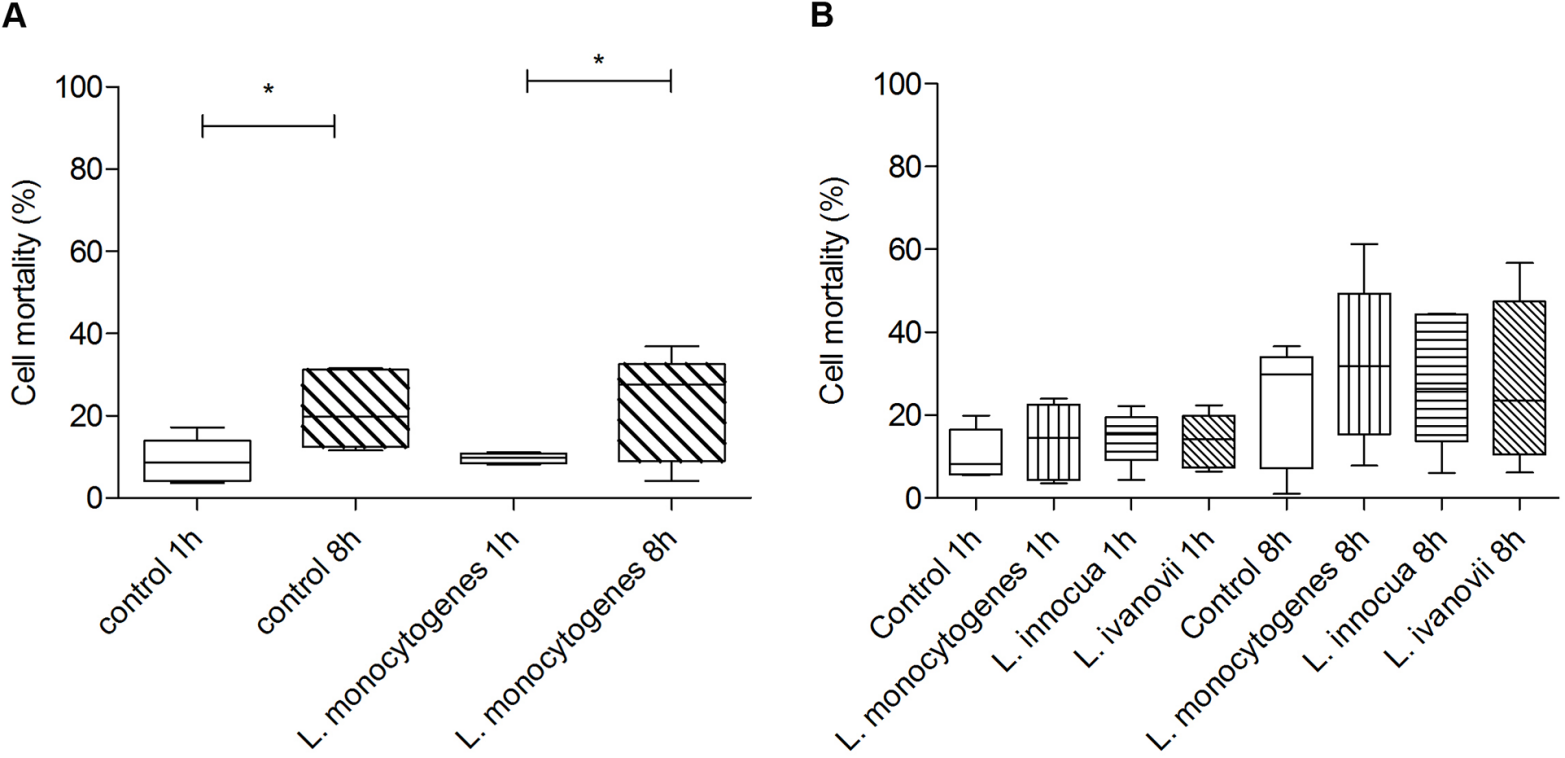
Cell mortality rate of bovine trophoblasts infected with *Listeria* spp. **(**A) Cell mortality was analyzed by measuring the LDH released in the supernatant of CAM explants infected with *L*. *monocytogenes* or non infected controls at 1 and 8 hpi. (B) Cell mortality was analyzed by measuring the LDH released in the supernatant of explants uninfected or infected with *L*. *monocytogenes*, *L*. *innocua*, or *L ivanovii* at 1 and 8 hpi. The value of 100% cell mortality was calculated as optical density measured for LDH of total trophoblastic cells from explants lysed with mix substrate Cytotox kit Non-Radioactive Cytotoxicity 96 Assay. Results are presented as median and range of the data of five independent experiments performed in triplicate (Mann Whitney, * p <0.05).

Considering that both *L*. *monocytogenes* and *L ivanovii* were able to multiply in bovine trophoblasts, we investigated whether these bacteria were capable of inducing lyses of trophoblasts. Cell mortality was determined by measuring LDH release in the supernatant of infected cells as compared to uninfected controls at 1 and 8 hpi. Interestingly, there was no difference between cell death of uninfected and infected with *L*. *monocytogenes* infection (Fig 4A), indicating that the bacterial infection did not induce cell death in infected trophoblasts even at time points when the bacteria is undergoing intracellular multiplication. However, an increase in the rate of cell death was observed at 8 hpi when compared to 1 hpi in both *Listeria*-infected and uninfected controls. This increase in LDH at 8 h is likely due to a limited viability of the CAM explants in culture. However, more than 60% of the cells remain viable at 8 h in culture ensuring the suitability of this model. Trophoblastic cell mortality was further evaluated in CAM explants infected with *L*. *monocytogenes*, *L*. *innocua*, or *L*. *ivanovii* at 1 and 8 hpi, and no differences were observed between these bacterial species, which supports the notion that *Listeria* intracellular multiplication does not induce trophoblastic cell lysis at times earlier than 8 hpi (Fig 4B).

### Expression of pro-inflammatory genes in bovine CAM explants infected with *Listeria* spp.

**Fig 5:**
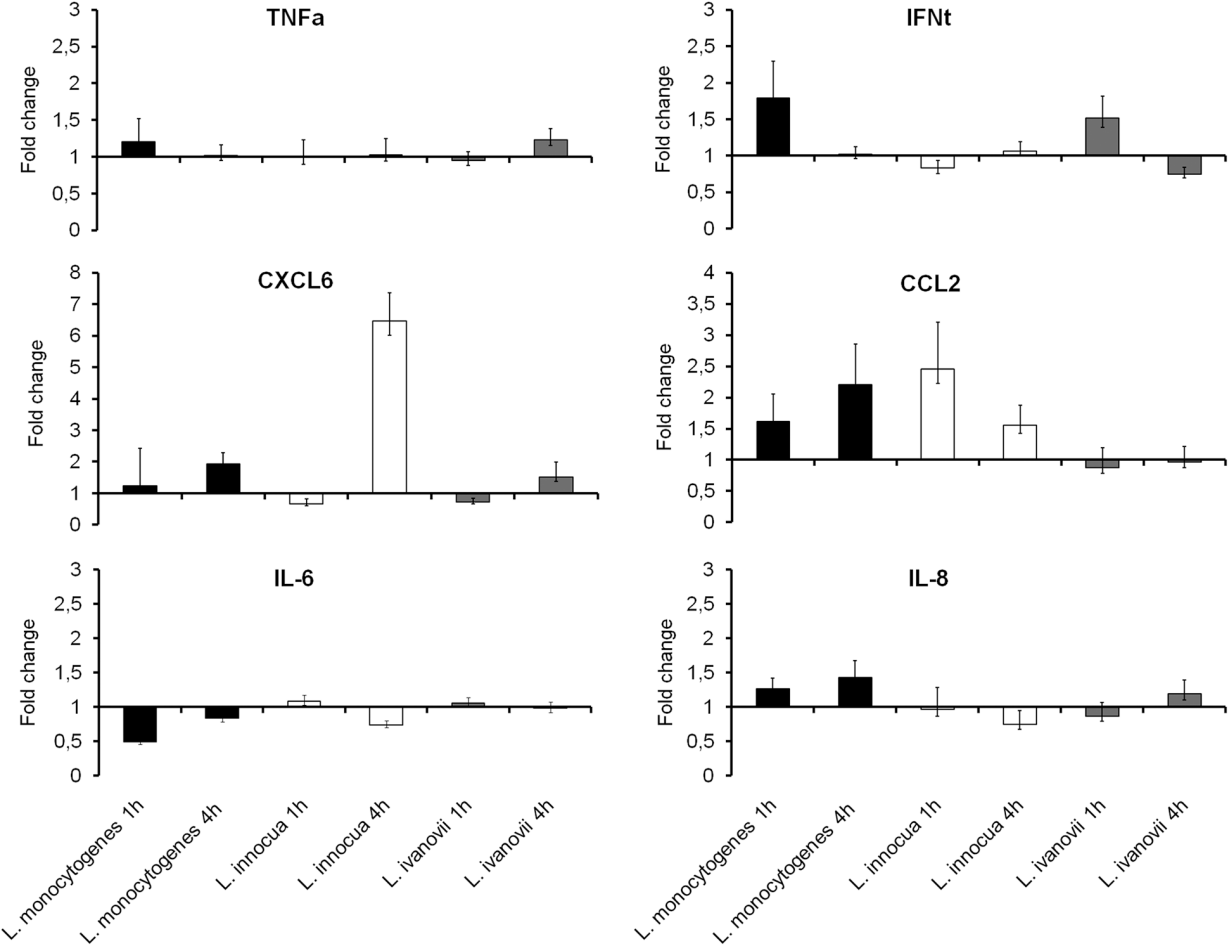
Proinflammatory gene expression of bovine CAM explants infected by *Listeria* spp. at 1 and 4 hpi. The explants were inoculated with 1x 10^6^ CFU/mL of *L*. *monocytogenes*, *L*. *innocua*, or *L*. *ivanovii* and total RNA extracted for quantification of mRNA by qPCR. Ct values were normalized for GAPDH and the data expressed as fold change compared to uninfected explants. Data represent the geometric mean and standard error of six independent experiments (ANOVA, * p <0.05).

Considering that *L*. *monocytogenes* and *L*. *ivanovii* were able to invade and multiply in bovine CAM explants without causing significant trophoblastic cell death up to 8 hpi, next we assessed whether *Listeria* spp. induced a pro-inflammatory transcription profile during the course of infection. Six bovine placentas were used to prepare CAM explants (three per treatment) that were no-infected or infected with *L*. *monocytogenes*, *L*. *innocua*, or *L*. *ivanovii*. Transcription of pro-inflammatory genes was assessed by qPCR at 1 and 4 hpi. A significant increase in transcription of CXCL6 was observed in trophoblastic cells infected with *L*. *innocua* at 4 hpi. None of the other genes had any significant change in transcription during the course of infection (Fig 5).

## Discussion

In this study we demonstrate for the first time, that *L*. *monocytogenes* can invade and multiply in bovine trophoblastic cells of the second and third trimester of pregnancy, and that *L*. *monocytogenes* and *L*. *ivanovii*, two species that cause abortion in ruminants, had similar levels of invasion and intracellular multiplication in bovine trophoblasts, which contrasts to the non-pathogenic *L*. *innocua* that is not capable of multiplying intracellularly in trophoblasts. These results support the notion that these two virulent species of *Listeria* spp. carry the virulence factors required for survival and multiplication in bovine trophoblastic cells, which may contribute to the occurrence of abortion that characterizes bovine listeriosis. The Listeriolysin O and ActA are known virulence factors required for bacterial multiplication in trophoblastic cells in mice (18). These two virulence factors are present in *L*. *monocytogenes* and *L*. *ivanovii* [12]. However, sphingomyelinase C, present only in *L*. *ivanovii* is important to intracellular multiplication in an epithelial cell line [9]. Genomic studies have shown differences between *L*. *monocytogenes* and *L*: *ivanovii* and suggest that these may be associated with stronger tropism of *L*. *ivanovii* to ruminants when compared to human hosts [9, 12]. Guillet et al. (2010) [6] demonstrated that *L*. *ivanovii* is more invasive than *L*. *monocytogenes* in MDBK but not in HeLa, which are bovine and human cell lines respectively. Although, we observed on average more than 0.5 log of intracellular *L*. *ivanovii* when compared to *L*. *monocytogenes* under the same conditions at all time points of infection, there were no statistically significant differences.

It has been reported that bovine abortion due to *Listeria* spp. infection occurs mostly during the last trimester of pregnancy [1, 2], our results demonstrated that there were no significant differences between trophoblasts of the second or third trimester of gestation in terms of *Listeria* invasion and intracellular survival. *L*. *monocytogenes*-induced fetal loss in women can occur at any stage of pregnancy [1]. It is thought that infection by *Listeria* sp. in cattle can cause fetal loss at any point in pregnancy, but embryonic loss is seldom recognized [1]. In addition, immunosuppression associated with pregnancy is recognized as a factor that favors *Listeria* infection [1, 5].

*L*. *innocua* is considered a non pathogenic bacterium [4], and it does not express several important virulence factors required for invasion and intracellular survival [18]. Indeed, our data demonstrated that *L*. *innocua* was able to invade bovine trophoblasts similarly to other pathogenic *Listeria* species, although *L*. *innocua* does not multiply intracellularly in bovine trophoblasts. Importantly, there were no previously reported evidences that *L*. *innocua* is capable of invading trophoblasts. *L*. *monocytogenes* invasion of human trophoblastic cells requires InlA and InlB [11]. This invasion factors are also required for pathogenesis *in vivo* in gerbils and mice expressing humanized E-caderin [19]. In contrast, invasion and dissemination of the placental infection may not require interaction of the internalins with E- caderin, likely occurring through a direct cell to cell invasion as observed in normal mice–i.e. lacking a humanized version of E-caderin [18, 21]. *L*. *innocua* is persists in placental tissues of pregnant mice up to 2 days, but it does not cause any change in the fetuses since it is unable to cross the placental barrier [24]. Furthermore, there are evidences that others cell receptors, in addition to E-caderin, may interact with other *Listeria* proteins promoting internalization of the bacteria into epithelial cells [13]. Additionally, bovine trophoblastic cells have phagocytic activity so it is possible that of *L*. *innocua* internalization may occur by phagocytosis [31], which is independent of InlA and InlB that is absent in *L*. *innocua* [11]. Internalization by phagocytosis has also been described in macrophages [10]. Tachibana et al. (2015) [32] demonstrated that macropinocytosis contributes to bacterial invasion into murine TG cell lines, and that this process may involve putative cell surface receptors in lipid rafts. Studies demonstrate that the PI3-K signaling pathway contributes to bacterial invasion in mouse and human trophoblastic cells [32, 33]. Additionally, MAPK signaling pathway may promote *L*. *monocytogenes* invasion into TG cell by activation of TLR2 [34] Thus, we hypothesize that in spite of being fully invasive in trophoblasts, the inability of *L*. *innocua* to multiply in trophoblastic cells, causes the self-limiting spreading to neighboring cells, and therefore fetal infection does not occur, leading to a non-pathogenic phenotype of this species.

Our results demonstrated that none of the *Listeria* species studied (i.e. *L*. *monocytogenes*, *L*. *ivanovii*, and *L*. *innocua*) caused trophoblastic cell death up to 8 hpi. *Listeria*-induced bovine trophoblastic cell death has not been previously evaluated. *Listeria* spp. induces cell death in infected human trophoblast [15, 35] as well as in other type cells [12], but *Listeria*-induced host cell death is observed at later time points of infection.

Bovine trophoblasts are epithelial cells that participate in placentation and maintenance of pregnancy. These cells have the ability to perform phagocytosis, invade tissues, and to produce cytokines such as IL-6 and IL-8 in response to infection [31, 36]. Cytokines such as TNFα, IL-6, IL-8 play an important role in the control of listeriosis by neutrophil recruitment in mice [22]. We have previously evaluated the cellular response of the bovine trophoblast exposure to heat killed *L*. *monocytogenes*, and we observed the induction of neutrophil chemokines after 12 h of stimulation [37], but the effect of the internalization of bacteria on cell response has not been evaluated. Thus, we evaluated the expression of pro-inflammatory genes, cytokines TNFα, IL-6, IL-8 IFN-τ and chemokines CXCL6 and CCL2 by pathogenic *Listeria* spp. infection in bovine trophoblasts, and yet there was not an increase in the expression of these genes until 4 hpi. Although Jung et al. (1995) [38] have shown that *L*. *monocytogenes* stimulates the transcription of pro-inflammatory genes in colon epithelial cells 4 hpi.

Interestingly, *L*. *innocua* was the only bacterium to induce expression CXCL6 at 4 hpi. Although *L*. *monocytogenes* induces pro-inflammatory cytokines in trophoblastic cells [14, 39] and other cell lines [10], Santoso et al. (2012) [14] have shown that it may inhibit expression pro-inflammatory cytokines as IL-6 in human cytotrophoblast cell line. Other studies demonstrate that *L*. *monocytogenes* are able to evade the innate immune response by inhibiting early expression of pro-inflammatory genes through deacetylated peptidoglycan [40] or by internalin C (InlC), present in *L monocytogenes* and *L*. *ivanovii*, which impairs activation of NF-κB pathway [41]. Previously, we demonstrated that *Brucella abortus*, other important abortive agent for cows, also delays initial pro-inflammatory responses in bovine trophoblasts [26].

In conclusion, this study has demonstrated for the first time that *L*. *monocytogenes* and *L*. *ivanovii* are able to invade and multiply intracellularly in bovine throphoblasts, whereas *L*. *innocua* invades, but it is unable to multiply in the intracellular environment. CAM explants are a suitable model to expand the knowledge on the molecular pathogenic mechanisms of *Listeria* spp. that are involved in *Listeria*-induced abortion in ruminants.

## Supporting information

S1 Fig Representative CT results from one bovine CAM explant set up in customized Taqman gene expression system plate. (TIF)
