# Supplementary figures and images for: Comparative experimental infection of Listeria monocytogenes and Listeria ivanovii in bovine trophoblasts
Source: PLoS One. 2017 May 3;12(5):e0176911. doi: 10.1371/journal.pone.0176911 (PMC5415186; doi:10.1371/journal.pone.0176911)

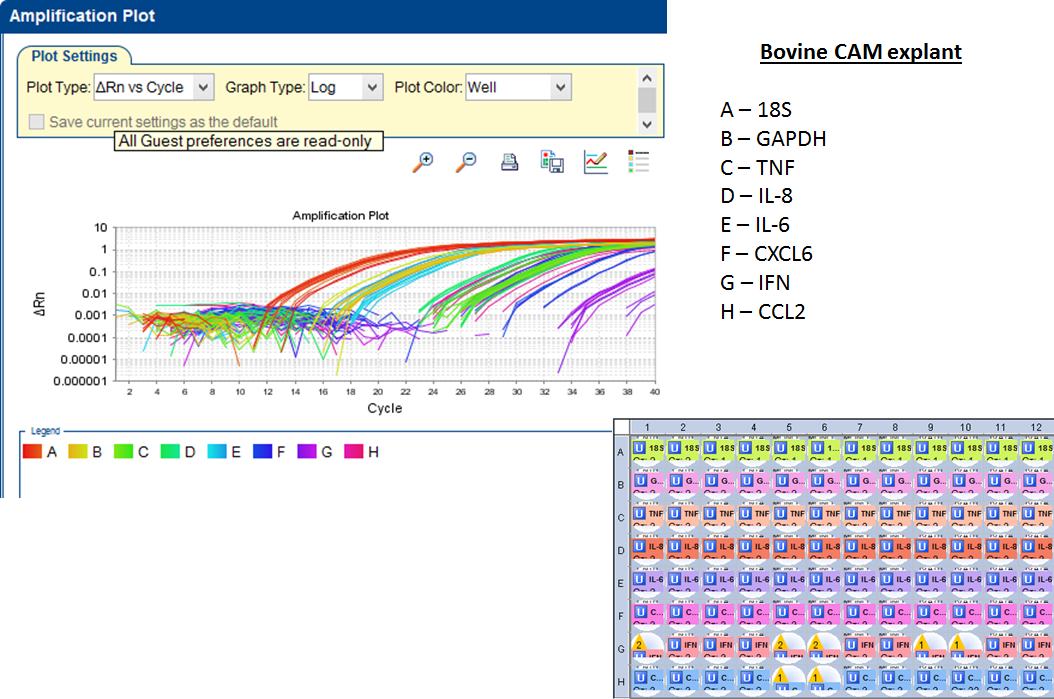

Supplement: S1 Fig — (TIF) [file pone.0176911.s001.tif]
